# Cognitive enhancing effects and anticholinesterase activity of stem bark and leaf extracts of *Prunus africana*

**DOI:** 10.1016/j.heliyon.2022.e12289

**Published:** 2022-12-17

**Authors:** David N. Ngai, Cromwell M. Kibiti, Mathew Piero Ngugi

**Affiliations:** a Department of Biochemistry, Microbiology and Biotechnology, School of Pure and Applied Sciences, Kenyatta University, P. O. Box 43844-00100, Nairobi, Kenya; b Department of Pure and Applied Sciences, Technical University of Mombasa, P. O. Box 90420-80100, Mombasa, Kenya

**Keywords:** Alzheimer's disease, Cognition, Passive avoidance task, Anticholinesterase, Acetylcholine

## Abstract

Alzheimer's disease is ranked among the top five causes of death for old people. Globally, it is approximated that there are 7.7 million new cases of Alzheimer's disease per annum and it is expected that by the year 2050, as many as 1.5% of people will be victims of Alzheimers or other types of dementia. Currently there is no cure for Alzheimer's disease and the conventional therapeutics agents available either have low efficacy or are associated with serious side effects. In the current study, *in vivo* cognitive advancing and anticholinesterase effects of crude methanol extracts of stem bark and leaf of *Prunus africana* were investigated in scopolamine treated mice. Passive avoidance task was used to evaluate cognitive enhancing effects of the two plant extracts. Donepezil was used as the standard drug. Scopolamine butylbromide (5 mg/kg bw) was administered intraperitoneally to induce Alzheimer's disease in mice during the study. A completely controlled randomised experimental design was employed in the current study. The two extracts displayed significant anticholinesterase activities and improved cognition in a dose dependent fashion as indicated by escape latency trends. From the current study, it is concluded that methanol extracts of stem bark and leaf of *P. africana* contain phytochemicals with anticholinesterase activity and cognitive enhancing effects in scopolamine treated mice. The study therefore supports use of leaf and stem bark extracts of *P. africana* for management of dementia by traditional herbal practitioners.

## 1 Introduction

Alzheimer's disease (AD) is caused by deficiency of a neurotrasnsmitter substance, acetylcholine, in the synaptic clefts. This deficiency compromises the functioning of cholinergic system which is a key component of memory development, learning and other cognitive processes in general (Vandesquille, 2011). Acetylcholine deficiency results from its reduced synthesis in the presynaptic knobs, increased degradation by cholinesterase at the clefts or insensitivity of muscarinic receptors to the neurotransmitter substance. Therefore, one of the strategies to ameriolate AD involves administration of cholinesterase inhibitors (AChEI) such as donepezil and galantamine among others (Kim et al., 2007).

Associative learning is a form of conditioning and is based on the theory that behaviour can be modified through repeated stimulus-response cycles. Thus, behaviour can be learned or unlearned as guided by the response it generates. This forms the basis of behavioural training in animals, including man (Bunadri et al., 2013). In the current study, mice were conditioned to associate pain with the dark compartment in passive avoidance task (PAT). The present study analysed brain cholinesterase (AChE) activity to determine the level of central cholinergic activity, which is related to levels of cognitive function, in plant extract treated amnesic mice.

Scopolamine impairs memory and learning via several mechanisms including antagonising cholinergic nervous transmission, stimulation of oxidative damage, reduction of brain-derived neurotrophic factor (BDNF) lelvels, phosphorylation of cAMP response element-binding protein and extracellular signal regulated kinase (ERK) which regulates development and consolidation of long-term memory and synaptic functions (Li et al., 2010; Um et al., 2018). Also, scopolamine produces a delirium-like condition in both animals and human (Qiu et al., 2016) by dysregulating cholinergic signals in the brain and antagonising M2 muscarinic receptors (Rubio et al., 2007). A substantial decline in cholinergic activity is the first pathophysiological indicator of AD (Hampel et al., 2018). This informed the choice of scopolamine to mediate cognitive deficiency with symptoms similar to those observed in AD patients.

Plants are a promising source of AD therapy because they are rich in phytocompounds with therapeutic effects against many ailments and, probably, Alzheimer's disease. Some phytochemicals have therapeutic effects due to their anti-AChE, anti-inflammatory or antioxidant activities (Yoo and Park, 2012). Others have metal-chelating activities, inhibit Aβ aggregation, or prevent hyperphosphorylation of tau proteins (Simunkova et al., 2019). Consequently, such plants are highly sought after due to these neuroprotective properties (Kim et al., 2007).

Presently, no cure is established for treatment of AD. The conventional medicines in the market are comprised of limited number of bio-actives resulting in insignificant efficacy and have many serious adverse effects. Fortunately, there are plant extracts that have proved to be relatively more potent due to enhanced synergistic effects resulting from high multiplicity of their constituent phytochemicals (Rubio et al., 2007). The present study aimed at investigating efficacy of *P. africana* stem bark and leaf extracts in alleviating cognitive deficiencies in scopolamine-induced mice models. The plant is broadly distributed in Africa and Kenya and is used for management of many ailments, including AD.

## 2 Materials and methods

### 2.1 Sampling and preparation of plant materials

Fresh stem barks and leaves of *Prunus africana* were sampled from ten mature trees at random at Kereita forest, Kiambu County (latitude -0.999967/S0º59881°; longitude 36627105/E36º3737.577°, Kimende 00221, Kenya). The botanical identity of *P. africana* (Hook, f.) was confirmed by a taxonomist at the National Museums of Kenya (NMK) herbarium under voucher specimen number DNN002. The plant samples were separately dried under shade at room temperature (25±1 °C) until they were dry and pulverized into a fine powder by use of an electric mill. The powders of the two materials were then separately packaged in clean, dry, sealed khaki bags and stored at room temperature (25±1 °C) until the time of extraction.

### 2.2 Extraction

The ground plant material (500 g) were separately mixed with one litre of methanol and incubated at room temperature with regular swirling for 24 h. The resulting broth was decanted into a sterile dry 5 L glass conical flask and poured into another sterile dry 5 L glass conical flask through a piece of clean cotton wool to sieve out the powdered debris. The resultant solution was filtered under pressure using suction force. The collected filtrate was concentrated under vacuum pressure using rotary evaporator at 40 °C and kept at 4 °C ready for use in subsequent studies. The percentage yields of the extracts were computed following the formula provided by Leal et al. (2011).

$$Percentage\text{ ​}yield=\frac{Mass\text{ ​}of\text{ ​}extract\text{ ​}obtained(g)}{Mass\text{ ​}of\text{ ​}powder\text{ ​}extracted(g)}\times 100$$

### 2.3 Sample preparation for LC-MS analysis

A 1 g sample of each extract was dissolved in 1 mL (90:10 MeOH, ddH_2_O), vortexed for 10 s, sonicated for 30 min then centrifuged at 14000 rpm for 10 min at 4 °C and supernatant analyzed using UPLC-MS/MS (0.1 μL).

### 2.4 Liquid chromatography—mass spectrometry (LC-MS) analysis

ACQUITY UPLC I-class system (Waters Corp., Milford, MA) was used to carry out quantative phytochemical characterisation of the extracts. It was fitted with an ACQUITY UPLC BEH C18 column (2.1 Χ 150 mm, 1.7 μm particle size; Waters Corp., Wexford, Ireland, oven temperature of 45 °C). The autosampler tray was conditioned to 5 °C. The elueting solvent system contained water (solvent A) and methanol (solvent B) both acidified with 0.01% formic acid. The gradient system applied was 5% B, 0–2 min; 40% B, 2–4 min; 40% B, 4–7 min; 60% B, 7–8.5 min; 60% B, 8.5–10 min; 80% B, 10–15 min; 80% B, 15–19 min; 100% B, 19–20.5 min; 100% B, 20.5–23 min; 95% B, 23–24 min and 95% B, 24–26 min. The flow rate was maintained at 0.2 mililiters per minute. The UPLC was interfaced with an electrospray ionization (ESI) (Waters Xevo TQ-S) which was run in a full scan MS with positive ionization state. The m/z range for acquiring data was 40–2,000 with a 0.5 kV capillary voltage, 30 V sampling cone voltage, 150 °C source temperature and 120 °C desolvation temperature. Nitrogen desolvation flow rate was 800 L/h.

### 2.5 Experimental animals

Healthy male Swiss albino mice, 35–42 days old and weighing 25–30 g were acquired from Jomo Kenyatta University of Science and Technology, Juja, Kenya. They were housed in propylene cages (5 mice per cage) at 25 ± 1 °C and 12-hour dark/light period. The animals were provided with uncontrolled access to rodent pellets and water *ad libitum* throughout period of study. The animals acclimatized for seven days prior to commencement of experiments. Authorization for this study was obtained from NACOSTI (License No: NACOSTI/P/19/150).

### 2.6 Experimental design

This study used a completely randomised design. Thirty mice were randomly assigned to 6 groups, each comprising of five animals and given the following treatment; Group I (Normal control mice) were administered with normal saline orally daily followed by intra-peritoneal injection with normal saline on alternate days for ten days. Group II (Negative control mice) were treated with normal saline orally daily followed by intra-peritoneal injection with scopolamine on alternate days for ten days. Group III (Positive control mice) were given donepezil (DNP) orally daily followed by intra-peritoneal injection with scopolamine on alternate days for ten days. Group IV (Experimental group 1) were given plant extract at a dose of 25 mg per kg bw daily followed by intra-peritoneal injection with scopolamine on alternate days for ten days. Group V (Experimental group 2) were given the extract at a dosage of 50 mg per kg bw daily followed by intra-peritoneal injection with scopolamine on alternate days for ten days. Group VI (Experimental group 3) were given the plant extract at a dose of 75 mg per kg bw daily followed by intra-peritoneal injection with scopolamine on alternate days for ten days (Table 1). All treatment volumes were 0.1 ml.

**Table 1:** Summary of treatment schedule.

Group	Category	Therapy
I	Normal control	Normal saline
II	Negative control	Normal saline + Scopolamine
III	Positive control	DNP + Scopolamine
IV	Experimental group 1	25 mg/kg bw extract + Scopolamine
V	Experimental group 2	50 mg/kg bw extract + Scopolamine
VI	Experimental group 3	75 mg/kg bw extract + Scopolamine

The anticholinesterase drug, donepezil (0.5 mg/kg bw in normal saline), was used as the reference drug. All the mice except the ones in the normal control group were intraperitoneally administered with scopolamine, (5.0 mg/kg bw in normal saline) on alternate days. The plant extract doses used were constituted as 25 mg, 50 mg and 75 mg/kg body weight. Plant extract, reference drug or normal saline were administered by oral gavage intragastrically daily for 10 days, 30 min prior to scopolamine or normal saline injection (Table 1). Thirty minutes duration was allowed between injection and behavioural tests.

### 2.7 Passive avoidance task protocol

A modified passive avoidance task as described by Ogren et al. (2008) was utilized to assess effects of *P. africana* extracts on associative learning in scopolamine treated mice. Equipment for passive avoidance task comprised a box partitioned into a lighted cubicle and a dark cubicle of similar dimensions (25 cm × 20 cm × 20 cm). Partition of the box had a square sliding door measuring 5 cm × 5 cm. The floor of the dark compartment was lined with wire mesh, connected to AC power source. A galvanometer was installed in the system to step down electric current to avoid causing harm to animals. The system produced a mild foot shock when circuit was completed using a push-switch button. The following three procedures; pre-trials (habituation), training and testing comprised the PAT protocol.

### 2.8 Pre-trials

In the current study, term “pre-trial” is used to refer to test on escape latencies carried out following the end of acclimatization period. Pre-trial tests were aimed at determining the entry behaviour of mice before they were used in study. Only mice that recorded a latency time of 150 s and below (50% score) were considered fit and were used in the study.

On pre-trials day, each animal was put in the lighted chamber of passive avoidance box (PAB), facing opposite the dark side, and allowed to freely roam about for 10 s. Partition door was then quietly raised and the animal was allowed to continue exploring freely. After the mouse fully entered the dark chamber with four paws, the door was closed, and latency to enter was documented using a video camera (supplementary videos V1–V6). The mouse was confined in the dark compartment for 10 s before it was returned to its home cage.

### 2.9 Training

The purpose of training was to condition the mice to associate the dark compartment, which is otherwise considered safe, with pain. This fear aggravated memory in mice increased the latency to move to the dark chamber of PAB. Briefly, each mouse was positioned in the lit chamber facing opposite the dark chamber, and allowed to move about freely for 10 s. The sliding door was then quietly opened. After the mouse entered the dark chamber with all the limbs, the sliding door was shut and latency to enter was captured using a video camera. Three seconds after closing the door, foot electro-shock (0.5 mA) was introduced for 1 s. Twenty seconds after the foot shock, the mouse was returned to its housing cage.

### 2.10 Testing

Testing was done at the end of the treatment period to assess the cognitive function and memory in mice. Briefly, on the tenth day, each animal was placed in the lit chamber of PAB, facing away from the dark side. After 10 s, the sliding door was raised to allow entry of mouse into the dark chamber. When mouse crossed into the dark cubicle with all limbs, the door was shut and latency to cross over documented using video camera. The animal was then returned to home cage.

### 2.11 Cholinesterase assay protocol

After passive avoidance task, cholinesterase activity was assessed using a colorimetric procedure described by Ellman et al. (1961) with slight modifications. Briefly, the mice were euthanized immediately following the end of PAT and whole brains dissected out. Each brain tissue was homogenized using tissue homogenizer in 0.5 ml sodium phosphate buffer (pH 7.4) under ice conditions. Subsequently, 0.1 ml phosphate buffer (pH 8.0) was poured into the tissue homogenate then centrifuged for 10 min at 3000 rpm under ice conditions.

A mixture of 170 μl of 4% DTNB, 470 μl sodium phosphate (0.1 M, pH 8.0) and 66 μl of supernatant was incubated for 5 min at 37 °C. Thereafter, 280 μL containing 1mM acetylthiocholine iodide substrate were added to the mixture and incubated for 3 min at 37 °C, after which optical density (OD) was measured at 410 nm.

### 2.12 Statistical analysis

MassLynx version 4.1 SCN 712 (Waters) was used to acquire data on phytochemistry. Potential compounds were assigned after mass spectrum for each peak was generated, and identification of molecular ion peaks was done using adducts, common fragments and by use of literature from online databases (METLIN, ChemSpider). Authentic samples, where available, were also used to confirm identify of compounds through co-injections. Relative abundance of each constituent phytocompound was expressed as a percentage with peak-area normalization. The latency of each mouse to enter the dark compartment in PAT was read directly from a digital camera while in case of cholinesterase activity, data were obtained spectrophotometrically. Obtained data were entered into excel spread sheet and descriptive analysis carried out. Inferential analyses were carried out in Minitab statistical software, v19. Significant differences between group-means was tested by One way analysis of variance (one way ANOVA) after which Tukey's post hoc test was done for pair-wise comparison of means. Efficacy between stem bark and leaf extracts was compared using unpaired student t-test. Paired student t-test was used for comparison of latencies after induction with the ones after treatment in PAT. The results were presented as mean ± standard error of the mean. Means with p-value <0.01 were considered statistically different. Cholinesterase activity was computed using the following formula (Cheng and Tang, 1998);

$$AChE\text{ ​}Activity\text{ ​}=\frac{B}{\Delta T\times V}\times D$$

where: B= Concetration of choline from the Standard Curve (nmol/L), ΔT = Reaction time expressed as minutes, V= Volume of the sample pippeted into the reaction well (ml), D = Sample dilution factor.

## 3 Results

### 3.1 Phytochemical profile of methanol extracts of *P. africana*

**Figure 1:**
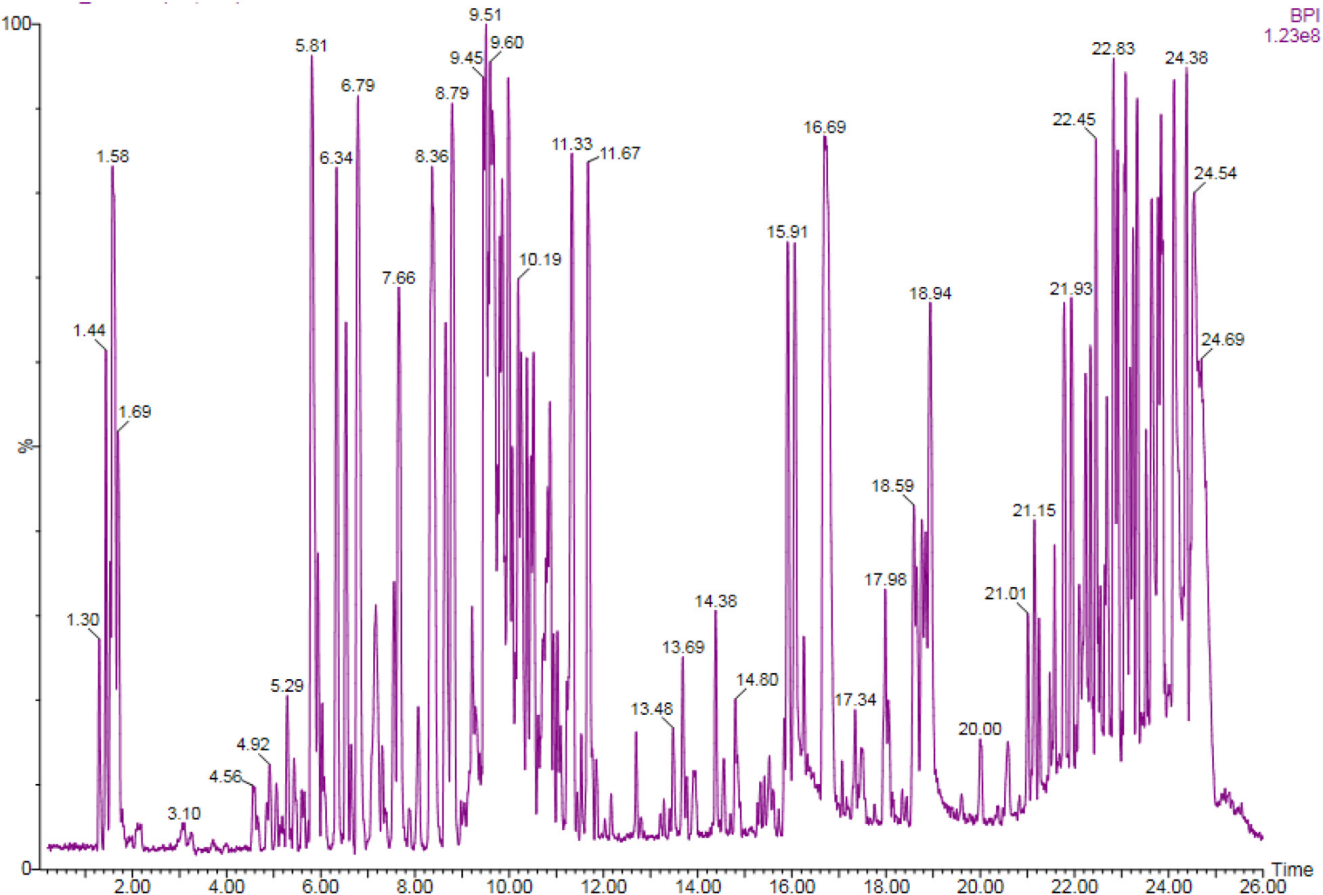
LC-MS chromatogram of methanol extract of stem bark of *P. africana* with retention time.

In the current study, percentage yield of methanolic stem bark extract was 4.719% while that of leaf extract was 8.23%. LC-MS analysis of the stem bark extract of *P. africana* identified an anthocyanin, Cyanidin-O-galactoside, as the most abundant constituent with percentage abundance of 20.34%. This was followed by chlorogenic acid (18.14%), quercetin (12.32%), quercetin 3,3′-dimethyl ether-4′-glucoside (10.26%), oleic acid (6.91%), beta sitostenone (6.82%) and catechin (6.23%) among others (Table 2). The full results including the chemical class of the phytochemical, retention time in UPLC, molecular formula and concentration in nanograms per gram (ng/g) of various phytocompounds present in the stem bark extract are summarised below (Table 2; Figure 1).

**Table 2:** Phytochemical profile of methanol stem bark extract of *P. africana* upon LC-MS analysis.

Rt	Compound Name	Molecular Formula	Chemical Class	Conc (ng/g)	Abundance (%)
1.44	Catechin	C_15_H_14_O_6_	Flavonoid	47.66	6.23
1.53	Prunasin	C_14_H_17_O_6_	Cyanogenic glucoside	18.91	2.47
1.58	Chlorogenic acid	C_16_H_18_O_9_	Polyhenol	138.74	18.14
1.69	Oleic acid	C_18_H_34_O_2_	Fatty acid	52.87	6.91
3.10	p-coumaric	C_9_H_8_O_3_	Polyphenol	0.57	0.07
4.85	Rutin	C_27_H_30_O_16_	Flavonoid	4.30	0.07
5.65	Zeatin	C_10_H_13_N_5O_	Cytokinin	5.31	0.69
8.39	Quercetin 3,3′-dimethyl ether-4′-glucoside	C_22_H_22_O_12_	Flavonoid	78.51	10.26
8.57	Apigenin 6-C-glucoside	C_26_H_28_O_14_	Polyphenol	0.07	0.01
8.79	Cyanidin-O-galactoside	C_21_H_21_ClO_11_	Flavonoid	155.63	20.35
8.98	Kaempferol	C_15_H_10_O_6_	Flavonoid	3.36	0.44
9.05	Luteolin	C_15_H_10_O_6_	Flavonoid	5.56	0.73
9.21	Apigenin	C_15_H_10_O_5_	Flavonoid	32.93	4.30
9.60	Quercetin	C_15_H_10_O_7_	Flavonoid	94.23	12.32
10.74	Campesterol	C_28_H_48_O	Phytosterol	11.84	1.55
10.86	beta-Sitostenone	C_29_H_48_O	Phytosterol	52.16	6.82
10.95	beta-Sitosterol	C_29_H_50_O	Phytosterol	19.06	2.49
11.02	Prunetrin	C_22_H_22_O_10_	Flavonoid	19.57	2.56
11.09	Ursolic acid	C_30_H_48_O_3_	Triterpenoid	11.93	1.56
11.22	Oleanolic acid	C_30_H_48_O_3_	Triterpenoid	11.67	1.53

Summary of compounds identified in stem bark extract of *P. africana* with their relative concentrations (ng/g). RT: Retention time (minutes).

**Figure 2:**
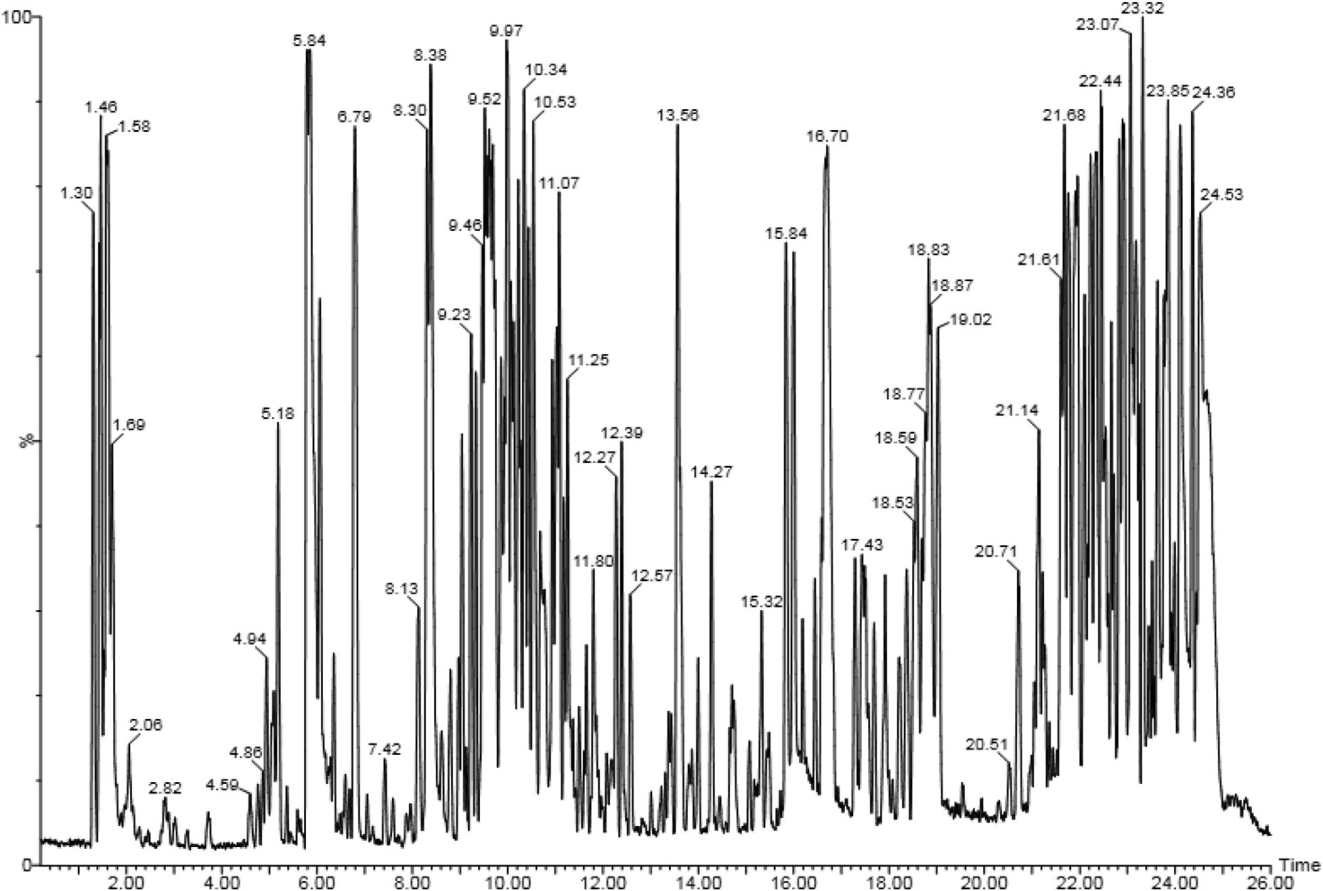
LC-MS chromatogram of methanol extract of *P. africana* leaf with retention time.

On the other hand, LC-MS analysis of methanol extract of leaf of *P. africana* identified a flavonoid, Quercetin 3,3′-dimethyl ether-4′-glucoside as the most abundant phytochemical in the extract with a percentage abundance of 17.35%. This was followed by oleic acid (8.91%), ursolic acid (8.27%), quercetin (8.04%), catechin (8.00%), chlorogenic acid (7.74%), apigenin (7.66%), prunetrin (7.04%) and octadecanoic acid (6.86%) among others (Table 3). The full results including the phytochemical class, retention time in UPLC, molecular formula and concentration in nanograms per gram (ng/g) of various phytocompounds are summarised below (Table 3; Figure 2).

**Table 3:** Phytochemical profile of methanol leaf extract of *P. africana* upon LC-MS analysis.

Rt	Compound Name	Molecular Formula	Chemical Class	RMM	conc (ng/g)	% Abundance
1.46	Catechin	C15H14O6	Flavonoid	290.26	70.39	8.00
1.53	Prunasin	C14H17O6	Cyanogenic glucoside	295.29	11.51	1.31
1.58	Chlorogenic acid	C16H18O9	Phenolic acid	354.31	68.09	7.74
1.61	Oleic acid	C18H34O2	Fatty acid	282.47	78.43	8.91
1.70	Octadecanoic acid	CH3(CH2)16COOH	Fatty acid	328.49	60.39	6.86
2.79	p-coumaric	C9H8O3	Polyphenol	164.16	2.11	0.24
4.86	Rutin	C27H30O16	Flavonoid	610.52	7.02	0.80
5.65	zeatin	C10H13N5O	Cytokinin	219.25	2.74	0.31
8.38	Quercetin 3,3′-dimethyl ether-4′-glucoside	C22H22O12	Flavonoid	478.40	152.74	17.35
8.61	Apigenin 6-C-glucoside	C26H28O14	Polyphenol	564.50	9.43	1.07
8.80	Cyanidin-O-galactoside	C21H21ClO11	Anthocyanin	484.83	21.63	2.46
8.97	kaempferol	C15H10O6	Flavonoid	286.23	18.01	2.05
9.04	Luteolin	C15H10O6	Flavonoid	286.24	47.94	5.45
9.23	Apigenin	C15H10O5	Flavonoid	270.03	67.39	7.66
9.61	Quercetin	C15H10O7	Flavonoid	302.24	70.79	8.04
10.76	Campesterol	C28H48O	Phytosterol	400.69	10.35	1.18
10.93	beta-Sitosterol	C29H50O	Phytosterol	414.71	46.44	5.28
11.03	Prunetrin	C22H22O10	Flavonoid	446.40	61.96	7.04
11.08	Ursolic acid	C30H48O3	Triterpenoid	456.70	72.79	8.27

Summary of compounds identified in leaf extract of *P. africana* with their relative concentrations (ng/g). RT: Retention time (minutes).

### 3.2 Comparison of the phytochemical profiles of methanol stem bark and leaf extracts of *P. africana*

**Figure 3:**
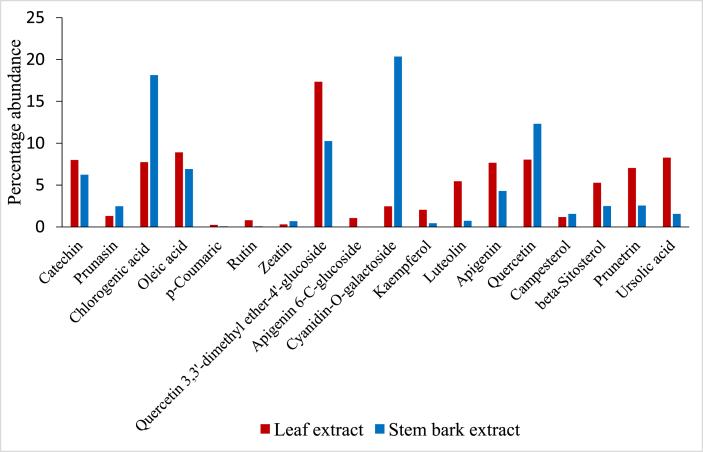
Comparison between abundance of phytochemicals in stem bark and leaf extracts.

The phytocompound profiles of methanolic extract of stem bark and leaf of *P. africana* revealed that the two extracts had a total of eighteen phytocompounds in common (Figure 3). However, beta-sitostenone and oleanolic acid were found in the stem bark extract but were absent in the leaf extract. On the other hand, octadecanoic acid was only present in the leaf extract (Table 2: Table 3).

The extract of *P. africana* stem bark proved to have higher abundance of prunasin, chlorogenic acid, zeatin, cyanidin-O-galactoside, quercetin and campesterol compared to the leaf extract (Figure 3). On the contrary, the leaf extract of proved to be richer in catechins, oleic acid, p-coumaric acid, rutin, quercetin 3,3′-dimethyl ether-4′-glucoside, apigenin, apigenin 6-C-glucoside, luteolin, beta-sitosterol, prunetrin and ursolic acid compared to the stem bark extract (Figure 3).

### 3.3 Effects of methanolic extracts of *P. africana* on escape latencies in PAT

The mean latencies for all mice in all studied groups except the normal control group were not significantly different after induction before administration of treatments (Tables 4 and 5).

**Table 4:** Effects of stem bark extract of *P. africana* on escape latencies in scopolamine treated mice in passive avoidance task.

		Latency time (seconds)	
Group	Treatment	After induction	After treatment
Normal control	Normal saline	278.80 ± 8.87^aA^	288.20 ± 7.27^aA^
Negative control	N.S/Scopolamine	52.00 ± 1.70^bA^	54.40 ± 1.17^eA^
Positive control	DNP/Scopolamine	53.60 ± 3.63^bA^	254.20 ± 3.60^bB^
Experimental 1	25 mg/kg bw extract/Scop	55.00 ± 3.86^bA^	186.20 ± 2.62^dB^
Experimental 2	50 mg/kg bw extract/Scop	52.80 ± 3.12^bA^	232.00 ± 2.39^cB^
Experimental 3	75 mg/kg bw extract/Scop	51.00 ± 3.81^bA^	263.40 ± 2.50^bB^

Values are presented as mean ± SEM per five mice. Means with different superscript lower case letters within the column and those with different superscript upper case letters along the row are significantly different according to one-way ANOVA followed by Tukey's post hoc test and paired t-test respectively (p < 0.01).

**Table 5:** Effects of leaf extract of *P. africana* on escape latencies in scopolamine treated mice in passive avoidance task.

		Latency time (seconds)	
Group	Treatment	After induction	After treatment
Normal control	Normal saline	282.40 ± 6.10^aA^	291.60 ± 4.37^aA^
Negative control	N.S/Scopolamine	52.40 ± 2.20^bA^	53.80 ± 2.44^eA^
Positive control	DNP/Scopolamine	52.20 ± 3.56^bA^	250.00 ± 4.12^bB^
Experimental 1	25 mg/kg bw extract/Scop	50.00 ± 1.70^bA^	180.60 ± 3.79^dB^
Experimental 2	50 mg/kg bw extract/Scop	52.60 ± 2.01^bA^	229.40 ± 2.52^cB^
Experimental 3	75 mg/kg bw extract/Scop	49.80 ± 2.56^bA^	256.80 ± 2.29^bB^

Values are presented as mean ± SEM per five mice. Means with different superscript lower case letters within the column and those with different superscript upper case letters along the row are significantly different according to one-way ANOVA followed by Tukey’s post hoc test and paired t-test respectively (p < 0.01).

**Figure 4:**
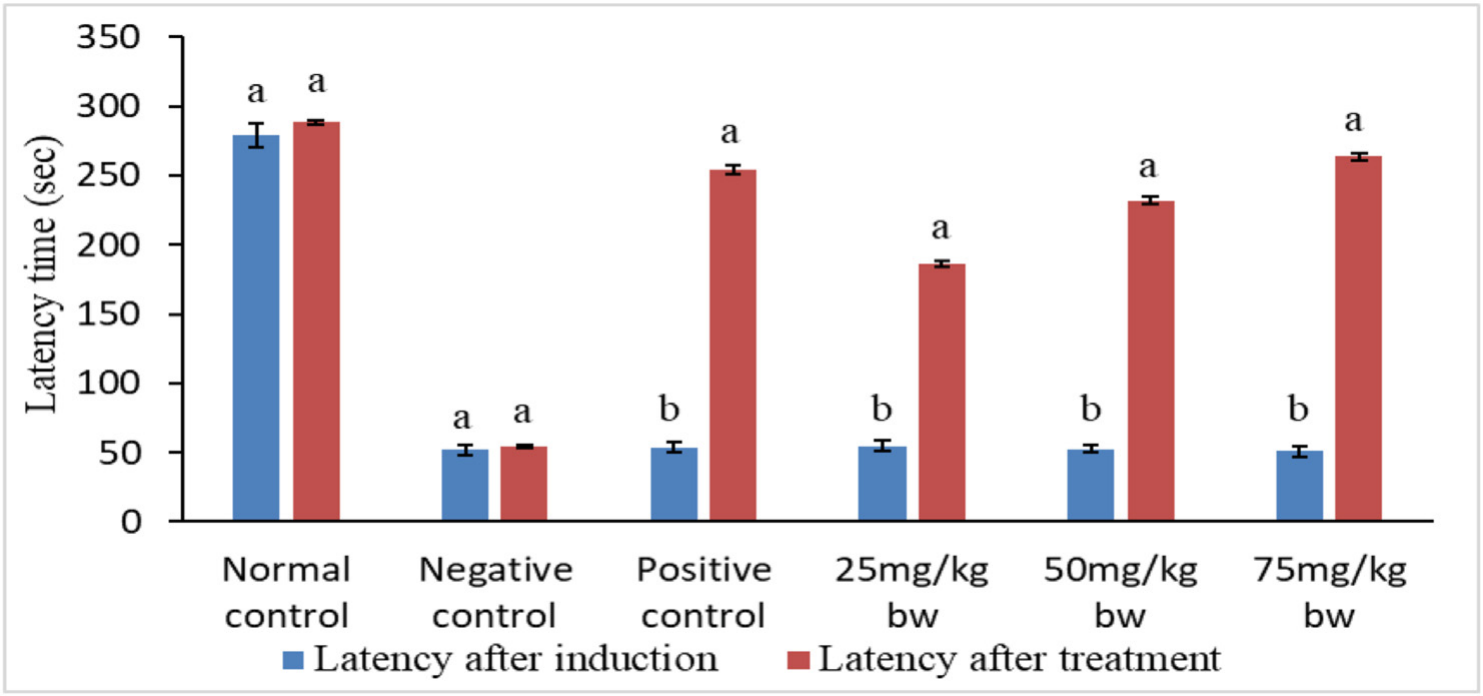
Comparison between latencies after induction and latencies after treatment with stem bark extract of *P. africana.* Bars with different lower case letters for each treatment are significantly different using paired t-test (p < 0.01).

**Figure 5:**
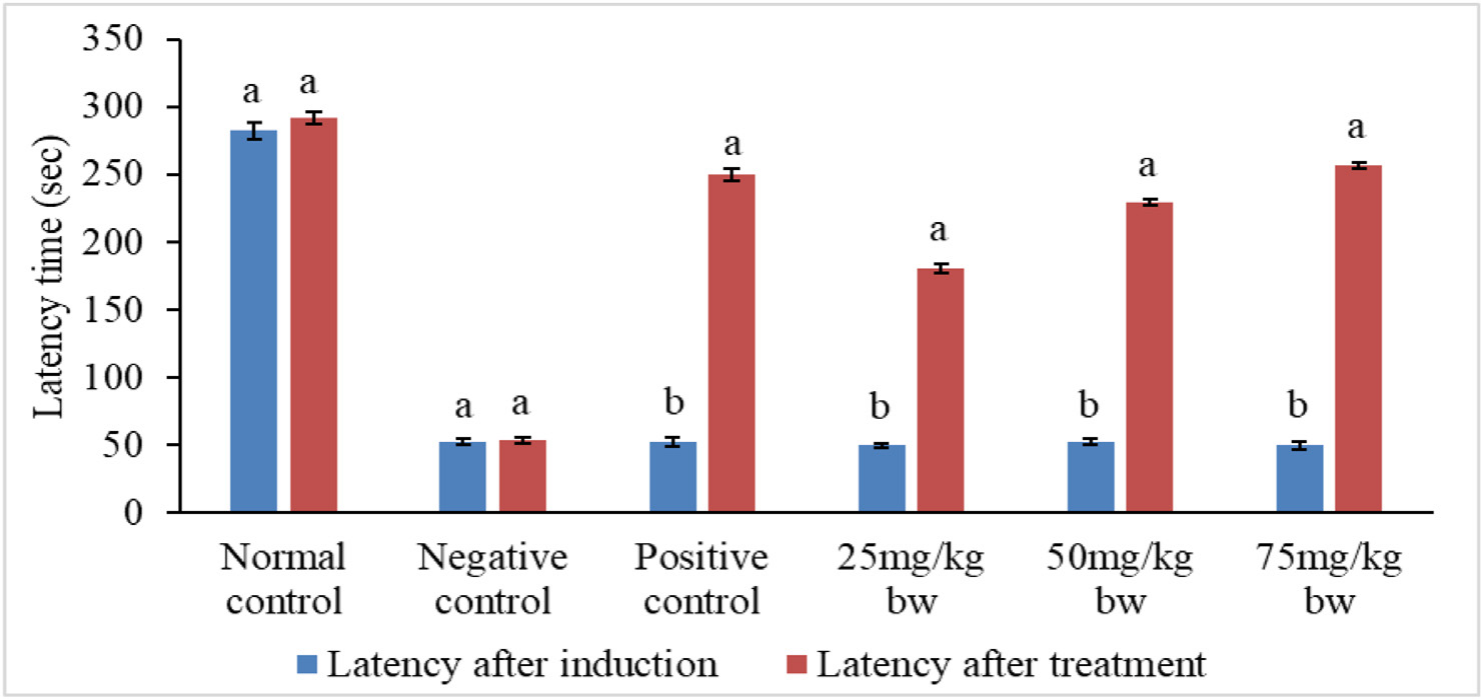
Comparison between latencies after induction and latencies after treatment with leaf extract of *P. africana.* Bars with different lower case letters for each treatment are significantly different using paired t-test (p < 0.01).

However, following treatments with reference drug and plant extracts, findings of the current study observed significant increase in latency time in groups III, IV, V and VI mice compared with latency time in group II (negative control) mice (p < 0.01) (Figures 4 and 5).

**Figure 6:**
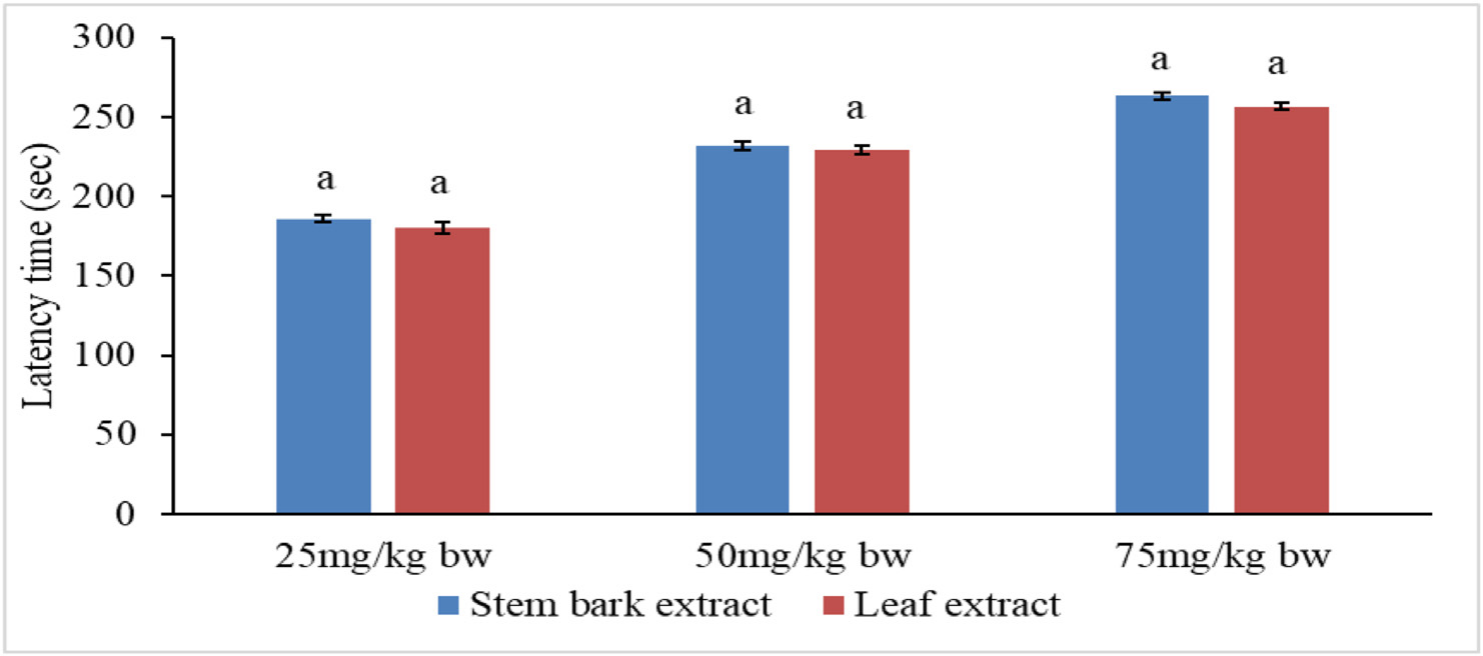
Comparison between the effects of stem bark and leaf extracts of *P. africana* on escape latencies. Bars with different lower case letters for each treatment are significantly different using unpaired t-test (p < 0.01).

Notably, the increase in escape latencies after treatment with stem bark and leaf extracts followed a dose dependent pattern. The activity of the standard drug was significantly higher than the extract activity at the doses 25mg and 50 mg/kg bw (p < 0.01). Nevertheless, concentration of 75 mg/kg bw of the extract was as effective as the standard drug (p > 0.01) (Tables 4 and 5). It was noted that stem bark and leaf extracts induced statistically alike (p > 0.01) escape latencies in passive avoidance task at the three dose levels tested (Figure 6).

### 3.4 *Ex vivo* anticholinesterase activity of methanolic extracts of *P. africana*

Mean cholinesterase activity in brains of all mice given stem bark extract and the positive control mice were significantly lower compared with mean cholinesterase activity in mice from the negative control group at the end of treatment period (Table 6). Inhibition of cholinesterase by stem bark extract followed a concentration dependent pattern (Table 6).

**Table 6:** Anticholinesterase activity of Stem bark extract of *P. africana* in scopolamine treated mice.

Group	Treatment	AChE activity nmol/min/ml
**Normal control**	Normal saline	10.45 ± 0.23^d^
**Negative control**	Normal saline/Scopolamine	15.21 ± 0.22^a^
**Positive control**	DNP/Scopolamine	12.78 ± 0.14^bc^
**Experimental 1**	extract 25 mg/kgbw/Scopolamine	13.19 ± 0.09^b^
**Experimental 2**	extract 50 mg/kgbw/Scopolamine	13.00 ± 0.15^b^
**Experimental 3**	extract 75 mg/kgbw/Scopolamine	12.13 ± 0.16^c^

Values pressed as mean ± SEM for 5 replicates. Means with different superscript letters are significantly different by one-way ANOVA followed by Tukey's post hoc test (p < 0.01).

Similarly, cholinesterase activities in homogenates from mice treated with reference drug and leaf extract were also significantly lower compared with those from negative control mice at the end of treatment period (Table 7). Inhibition of cholinesterase by leaf extract followed a dose associated pattern (Table 7).

**Table 7:** Anticholinesterase activity of leaf extract of *P. africana* in scopolamine treated mice.

Group	Treatment	AChE activity nmol/min/ml
Normal control	Normal saline	10.45 ± 0.23^d^
Negative control	Normal saline/Scopolamine	15.21 ± 0.22^a^
Positive control	DNP/Scopolamine	12.78 ± 0.14^c^
Experimental 1	extract 25 mg/kgbw/Scopolamine	13.67 ± 0.11^b^
Experimental 2	extract 50 mg/kgbw/Scopolamine	13.26 ± 0.16^bc^
Experimental 3	extract 75 mg/kgbw/Scopolamine	13.07 ± 0.14^bc^

Values pressed as mean ± SEM for 5 replicates. Means with different superscript letters are significantly different by one-way ANOVA followed by Tukey's post hoc test (p < 0.01).

**Figure 7:**
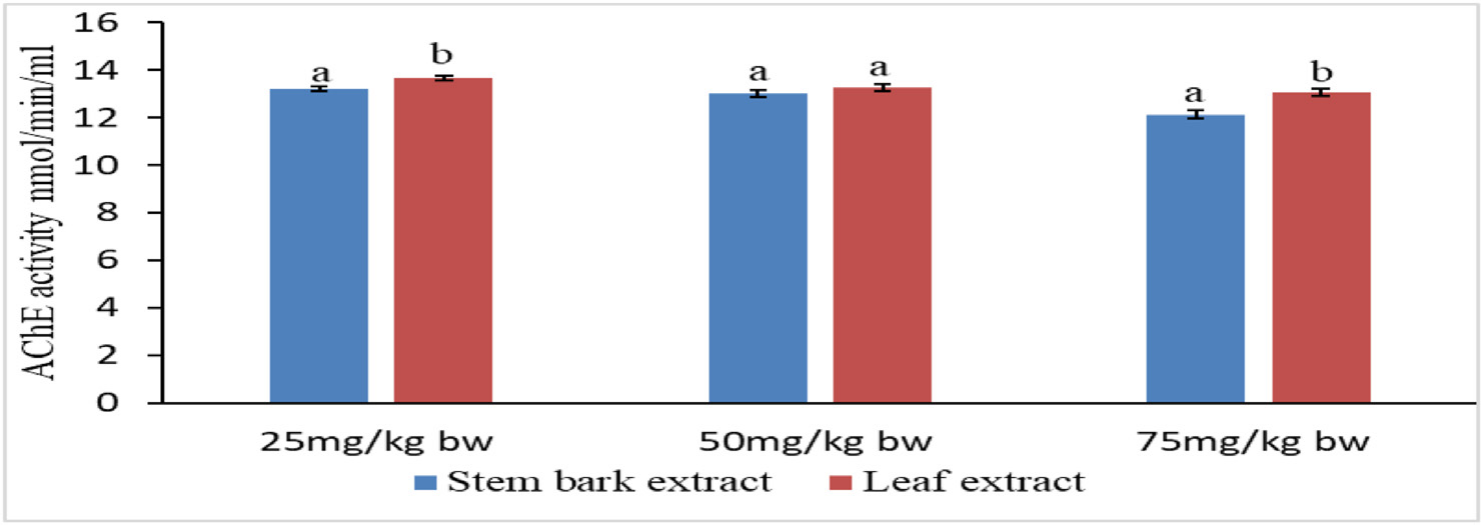
Comparison of cholinesterase activity of leaf and stem bark extracts of *P. africana.* Bars with different lower case letters for each treatment are significantly different using unpaired t-test (p < 0.01).

Leaf and stem bark extracts mediated statistically similar (p > 0.01) cholinesterase activity at dose of 50 mg/kg bw of *P. africana* extracts. However, at the lowest dose of 25 mg/kg bw and the highest dose of 75 mg/kg bw, stem bark extract was significantly more potent compared with leaf extract (Figure 7).

## 4 Discussion

The present study determined effects of leaf and stem bark extracts of *P. africana* on cognitive function and cholinesterase activity in memory-impaired AD mice models. Intraperitoneal administration of scopolamine induces sustained AD-like symptoms in rodents (Um et al., 2018). This route of drug administration is preferred because it does not trigger production of nitric oxide by endothelial cells, which can cause hypertension related complications, which are common when intravenous route is used. Nitric oxide is one of the reactive oxygen species that accelerate progression of Alzheimers disease (Ahmed et al., 2018) and therefore, can interfere with results of the study.

Findings from the current study show that scopolamine administration significantly enhanced AChE activity in the brain tissue of mice. Therefore, one of mechanisms of action of scopolamine may involve raising levels of AChE which ultimately results in accelerated degradation of acetylcholine at synaptic clefts. Scopolamine is reported to induce amnesia in young mice by impairing transcription of brain-derived neurotrophic factor which play a key role in control of memory development and plasticity, among other cognitive processes (Um et al., 2018). Ahmed et al. (2018) avers that there is a probability that scopolamine incursion also interferes with downstream effector mediators like cGMP which is required for long-term potentiation, leading to memory decline and neurobehavioral changes observed in AD patients.

After AD induction, there were no significant differences observed in escape latencies between the five groups of mice that received scopolamine treatment but their escape latencies dropped significantly compared to those of the normal control mice. The result concurs with those of a previous study by Sung et al. (1997). Scopolamine has been successfully utilized to develop animal models of Alzheimers disorder (Ahmed et al., 2018). Other chemicals used to induce AD in laboratory animals include D-galactose, which impairs learning and memory through several mechanisms, (including increasing oxidative stress, suppressing production of brain derived neurotrophic factor, hyperphosphorylation of extracellular signal regulated kinases and cAMP response element-binding protein), which mediate long term memory and other synaptic functions (Um et al., 2018).

The range of extract doses used in the current research was similar to the one used by Kim et al. (2006), when studying effects of *Cassia obtusifolia* seed extract in ameliorating learning and memory deficits in scopolamine-mediated dementic rats. Choice of dose range is also informed by concentration of various phytocompounds in plant tissues, which is influenced by prevailing weather conditions and quality of soils in terms of profile, structure and nutrient availability.

LC-MS analysis revealed presence of various phytochemicals with anticholinesterase activity, some coupled to antioxidant and antiinflamatory properties. The main classes of phytochemicals identified in the extracts included flavonoids, polyphenols, triterpenoids and fatty acids. Specific phytochemicals revealed included Cyanidin 3-O-beta-D-galactoside, Oleanolic acid, Chlorogenic acids, Ursolic acid, Quercetin, Vitexin and Isovitexin, among others. Previous studies have demonstrated that phytocompounds from medicinal plants can be converted into lead molecules that are effective against AD and other disorders (Elsawi et al., 2018).

Polyphenols and flavonoids are known to have appreciable antioxidant potential and several findings have validated their capability to cross blood-brain barrier and acknowledged their involvement in amelioration of AD via interference with some therapeutic targets like AChE and/or extracellular regulated kinase 2 (ERK2) (Elsawi et al., 2018). Apparently, mechanisms of action of extracts used against AD partially involve alleviation of oxidative stress, which in turn, decelerates progression of pathology (Khazdair et al., 2019).

The current study revealed valuable preliminary data on associative learning enhancing capacity of the two studied extracts through PAT. Mechanism of action of the phytocompounds found in methanolic extracts of *P. africana* was assessed by examining their inhibitory activity on cholinesterase in mice brain homogenates. Findings of the current study established that treatment of mice with stem bark and leaf extracts of *P. africana* attenuated memory decline activated by scopolamine in mice (Table 4.2 and 4.3). The memory enhancing capacity can be attributed to diverse bioactive phytocompounds contained in the extracts such as terpenoids and flavonoids whose anticholinesterase and antioxidant activities have been reported (Um et al., 2018). Efficacies of the two extracts were found to be similar probably because they had more or less similar phytochemical composition in terms of diversity. However, the proportions of various phytochemicals in stem bark and leaf extracts differed greatly.

The two studied extracts increased latencies to enter the dark compartment in a dose related manner in PAT and activity of AChE was inhibited significantly when the two extracts were administered. These findings demonstrated that the memory enhancing effects of *P. africana* can be associated with inhibition of AChE by various phytocompounds present in the extracts. No significant difference was found in potency between the two extracts as far as PAT results were concerned. However, more research is needed before conclusions are made because phytochemicals work in synergy and mere presence or absence of one or more of these bioactives, or a change in their proportions thereof, can drastically alter efficacy expectations of an extract (Elsawi et al., 2018).

Stem bark extract reduced cholinesterase activity significantly at all dose levels but no significant difference was found among potencies of the lower two doses. However, the highest dose of the extract was significantly more potent compared with the lower doses. All in all, effectiveness of any drug is not wholly dependent on administered quantity and in some cases lower doses have proved to be more effective than higher ones. Physical properties of phytochemicals, including solubility and polarity among others, are important because they influence bioavailability of a drug to some extent. Furthermore, the rate of degradation and stability of phytochemicals and pharmacokinetics of their secondary metabolites also determines their optimal doses in various medications (Yang et al., 2014).

Regarding cholinesterase activity, no significant differences was found between stem bark and leaf extracts at the level of 50 mg/kg bw dose. However, comparison between the extracts showed that stem bark extract was significantly better in reducing the cholinesterase activity at the lowest and the highest doses. The difference in phytochemical diversity and their relative abundances in the two extracts could explain the higher efficacy of stem bark extract compared with leaf extract at these dose levels. For instance, some phytochemicals such as oleanolic acid and sitostenone were present in stem bark extract but were absent in leaf extract while stearic acid was only present in leaf extract. Such variations definitely affect the relative potencies of the extracts to some extent.

Oleanolic acid acts by inhibiting IFN-γ, Nitric Oxide Synthase and cyclooxygenase-2 in rat macrophages. Oleanolic acid is also a strong inducer of phase-2 response through increase of heme oxygenase-1 and NADH-quinone oxidoreductase, which protect cells from free radicals and electrophile damage (Yang et al., 2014).

Similarly, sitostenone could have contributed to differences in potency between leaf and stem bark extracts as it was present in the stem bark extract only. Sitostenone has strong anti-inflammatory, neuroprotective and antioxidant activities that are keys in alleviating symptoms associated with Alzheimers disease (Abraham et al., 2001). In studies *in vivo*, it reduced AChE, TNF-α, and corticosterone activities and improved activity of antioxidant enzymes, thus contributing to neuroprotection. It has been pinpointed as a promising candidate for development of therapeutic agent for managing oxidative stress in various lifestyle diseases (Abraham et al., 2001).

The current study demonstrated a significant improvement (p < 0.01) in escape latencies in mice given the reference drug, stem bark and leaf extracts unlike in those in negative control group where escape latencies did not show any significant improvement by the end of study period. Furthermore, methanolic extract of *P. africana* stem bark, at all the three dosages, showed significant inhibition of cholinesterase enzyme as evidenced by differences between scores of experimental subjects and negative control subjects (Table 4.4). This finding can be ascribed to activity of polyphenols, flavonoids and other phytochemicals that possess anticholinesterase and antioxidant activities. As the experiment lasted 10 days, chances are that phytochemicals in the extracts negatively influenced expression of RNA and synthesis of AChE over the treatment period. This concurs with findings of a study reported by Uddin et al. (2016).

In addition, phenolic compounds, vitexin (apigenin) and isovitexin (apigenin 6-C-glucoside), which were more concentrated in leaf extract, have been demonstrated to have anti-Alzheimers, anti-diabetic and anti-inflammatory properties. Among them, isovitexin was singled out as the most potent inhibitor against AGEs generation, AChE, BChE, RLAR and HRAR activities, whereas vitexin was the most potent PTP1B inhibitor (Miller et al., 2004). In another previous study, isovitexin was reported as the only flavonoid that interacts with Tyr337 amino acid residue in AChE with a favourable docking score (−26.97 kcal/mol) due to its small size relative to that of bulkier glucosides (Colizzi, 2019). That points to possible mechanism of action through which the two extracts exert their influence on cholinesterase activity.

Quercetin, a flavonoid in higher concentration in the leaf extract compared with stem bark extract, protects integrity of neurones via activation of signalling pathways that control synaptic plasticity and long-term potentiation. It is also said to ameliorate neuroinflammation in AD (Olthof et al., 2003). This phytocompound directly or indirectly contributed to drastic recovery observed in AD mice models and the recorded anticholinesterase activity.

A polyphenol, chlorogenic acid is known to greatly improve scopolamine-induced decline in short-term memory. *Ex vivo* studies have showed that AChE activity was appreciably inhibited by chlorogenic acid in frontal cortex and hippocampus, indicating that it possesses antiamnesic properties (Orhan et al., 2004). Hippocampus is involved in memory acquisition, consolidation and cognitive learning.

Different organs of the same plant accummulates different phytocompounds in varying proportions and there are equal chances of the bioactives of interest being accumulated in any of the plant organs. Therefore, the fruits and roots of *P. africana* can be investigated in future studies. Moreover, use of nonpolar solvents or a combination of different solvents, would be recommended in future studies since different solvents and their combinations extract different phytochemicals.

A summary of phytochemicals with anticholinesterase, anti-inflammatory and antioxidant activity has been annexed as a supplementary table (Table S1). In addition, some video clips illustrating the apparatus and orientation of mice during behavioural tests have been added as supplementary materials (V1–V6).

## 5 Conclusion

The current study findings can be concluded by stating that methanolic stem bark and leaf extracts of *P. africana* contain several phytocompounds with anticholinesterase activity and cognitive enhancing effects in scopolamine treated mice. The study therefore supports use of stem bark and leaf extracts of *P. africana* for management of Alzheimers disease.

## Declarations

### Author contribution statement

David Nyaga Ngai: Conceived and designed the experiments; Performed the experiments; Analyzed and interpreted the data; Contributed reagents, materials, analysis tools or data; Wrote the paper.

Cromwell M. Kibiti; Mathew Piero Ngugi: Conceived and designed the experiments; Wrote the paper.

### Funding statement

This research did not receive any specific grant from funding agencies in the public, commercial, or not-for-profit sectors. The researcher used savings from his salary to fund the project.

### Data availability statement

Data associated with this study has been deposited at Kenyatta University Post Modern Library, Nairobi, Kenya under the accession number [Registration Number I84/38985/2017].

### Declaration of interest’s statement

The authors declare no competing interests.

### Additional information

Supplementary content related to this article has been published online at [URL].

## Appendix A Supplementary data

V1 Normal control (mouse 5) V1 Normal control (mouse 5)

V2 Negative control (mouse 1) V2 Negative control (mouse 1)

V3 Positive control (mouse 5) V3 Positive control (mouse 5)

V4 Experimental 1 (mouse 5) V4 Experimental 1 (mouse 5)

V5 Experimental 2 (mouse 2) V5 Experimental 2 (mouse 2)

V6 Experimental 3 (mouse 4) V6 Experimental 3 (mouse 4)
