# Supplementary material for: Cognitive enhancing effects and anticholinesterase activity of stem bark and leaf extracts of Prunus africana
Source: Heliyon. 2022 Dec 17;8(12):e12289. doi: 10.1016/j.heliyon.2022.e12289 (PMC9803689; doi:10.1016/j.heliyon.2022.e12289)
Supplement: Supplementary table (Table S1) [file mmc7.docx]

**Table S1 Neuroprotective properties of some Phytochemicals present in stem bark and leaf extracts of *P. africana***

| Phytochemical | Anticholinesterase activity | Antiinflammatory activity | Antioxidant activity |
| --- | --- | --- | --- |
| Catechin |  |  | x |
| Chlorogenic acid | x | x | x |
| Oleic acid |  | x |  |
| p-Coumaric |  |  | x |
| Rutin |  | x | x |
| Quercetin 3,3'-dimethyl ether-4'-Glucoside |  |  | x |
| Apigenin 6-c-glucoside | x | x | x |
| Kaempferol |  |  | x |
| Luteolin |  | x | x |
| Apigenin | x | x | x |
| Quercetin | x | x | x |
| Campesterol |  | x |  |
| Beta-sitostenone | x | x | x |
| Beta-sitosterol |  |  | x |
| Prunetrin |  | x | x |
| Ursolic acid |  | x | x |
| Oleanolic acid |  |  | x |

Summary of phytochemicals with anticholinesterase, anti-inflammatory and antioxidant activity. x = Activity present
